# Developmental Trajectories of Social Skills during Early Childhood and Links to Parenting Practices in a Japanese Sample

**DOI:** 10.1371/journal.pone.0135357

**Published:** 2015-08-12

**Authors:** Yusuke Takahashi, Kensuke Okada, Takahiro Hoshino, Tokie Anme

**Affiliations:** 1 Graduate School of Education, Kyoto University, Kyoto, Japan; 2 School of Human Sciences, Senshu University, Kawasaki, Japan; 3 Faculty of Economics, Keio University, Tokyo, Japan; 4 Faculty of Medicine, University of Tsukuba, Tsukuba, Japan; University of Tuebingen Medical School, GERMANY

## Abstract

This study used data from a nationwide survey in Japan to model the developmental course of social skills during early childhood. The goals of this study were to identify longitudinal profiles of social skills between 2 and 5 years of age using a group-based trajectory approach, and to investigate whether and to what extent parenting practices at 2 years of age predicted developmental trajectories of social skills during the preschool period. A relatively large sample of boys and girls (*N* > 1,000) was assessed on three social skill dimensions (Cooperation, Self-control, and Assertion) at four time points (ages 2, 3, 4, and 5), and on four parenting practices (cognitive and emotional involvement, avoidance of restriction and punishment, social stimulation, and social support for parenting) at age 2. The results indicated that for each social skill dimension, group-based trajectory models identified three distinct trajectories: low, moderate, and high. Multinomial regression analysis revealed that parenting practice variables showed differential contributions to development of child social skills. Specifically, Cooperation and Assertion were promoted by cognitive and emotional involvement, Self-control by social stimulation, and Assertion by avoidance of restriction and punishment. Abundant social support for parenting was not associated with higher child social skills trajectories. We found heterogeneity in developmental profiles of social skills during the preschool ages, and we identified parenting practices that contributed to different patterns of social skills development. We discussed the implications of higher-quality parenting practices on the improvement of child social skills across early childhood.

## Introduction

Social skills are defined as socially acceptable, learned behaviors that enable a person to interact effectively with others and to avoid socially unacceptable responses [1]. Developing social skills to enable successful relationships with others is one of the most important accomplishments in childhood. Conversely, when children fail to build social skills properly, or their skills do not function effectively in the early developmental phase, they subsequently display problem behaviors and experience later social maladaptation, school maladjustment, and poor academic performance [2, 3]. Therefore, it is important to identify children who are socially deficient, to provide intervention directed at improving their social skills, and to be proactive in taking adequate prevention measures.

Several large-scale longitudinal projects, including the National Head Start–Public School Early Childhood Transition Demonstration Project and the NICHD study of Early Child Care and Youth Development, have identified characteristics of children’s social skills development. For instance, children’s social skills, as perceived by parents, increased from kindergarten through third grade [4]. From kindergarten through fifth grade, children showed curvilinear social skill growth [5, 6]. Moreover, from adolescence to early adulthood, youth’s self-reported prosociality followed a horseshoe shaped trend: The overall level of prosociality declined until age 17, with a subsequent slight rebound until age 21 [7]. However, much less is known about the developmental trajectories of social skills during early childhood, ages 2 to 5. Children during this period are expected to begin demonstrating cooperation, assertive behavior, and self-control, while coping with greater environmental demands by teachers and peers [8]. Prior research has also indicated that prosocial behavior of many different sorts appears in early childhood [9, 10]. Thus, the preschool period of ages 2 to 5 is an important developmental stage in which children must acquire social skills to prepare for their upcoming school attendance and the expansion of their areas of social activity. From a socialization theory perspective [10], preschool-aged children learn with age how to be good; that is, they increase the frequency of prosocial behaviors. At the same time, there is evidence that children’s social skills become differentiated and thus show heterogeneity [11]. From this perspective, it would not be surprising if some individuals or groups demonstrate a declining (regulated) trend rather than a general upward trend. Indeed, some developmental profiles of prosocial behavior rated by teachers declined gradually from kindergarten to third grade [4], from age 6 to 12 [12], and from age 10 to 15 [13]. The first goal of the present study was to identify the heterogeneous developmental trajectories of social skills known to be components of prosocial behavior during early childhood by applying a group-based trajectory approach [14].

We expected that the number of longitudinal profiles of children’s social skills would be similar to trajectories found in prior research with different aged samples. Some researchers have reported a developmental trajectory for children’s prosocial behaviors, a broad construct of social skills. For instance, Côté, Tremblay, Nagin, Zoccolillo, and Vitaro [15] demonstrated that a three-latent class model best fit teachers’ ratings of children’s prosocial behavior throughout the school-age period. Kokko et al. [12] obtained two trajectories of prosocial behavior from ages 6 to 12 by means of a group-based trajectory method. Nantel-Vivier et al. [13] described cross-cultural developmental trajectories of prosocial behavior from ages 10 to 15 using a multi-informant approach. They identified three trajectory groups from self-ratings, five from mother-ratings, and three or four from teacher-ratings. Lamont and Van Horn [16] selected a three-class solution for four subscales of child social skills from kindergarten to third grade. Taken together, the results from these previous studies indicate that a group-based trajectory method, or growth mixture modeling, can be used to group prosocial behavior during school age into two to five separate latent classes. Therefore, we hypothesized that two to five latent classes could be identified in the developmental trajectory of social skills during early childhood, and that there would be at least one lower social skills group and at least one higher group, in addition to some middle-level groups.

The second goal of the present study was to investigate whether and to what extent parental and environmental factors predict trajectory group memberships. The previous studies reviewed above revealed only the grouping of developmental trajectories, suggesting that prosocial behavior during school age can be grouped into multiple trajectories. However, little is known about how the differences between the developmental trajectories should be characterized. After the developmental trajectories are identified, the factors that influence the developmental trajectories of social skills can then be identified. We expect that higher parenting quality at an early developmental stage acts as a growth-promoting factor. In fact, there exists an extensive body of literature showing that positive child-rearing environmental factors are associated with children’s social development [6, 17]. In a review of relevant research, Repetti, Taylor, and Seeman [18] concluded that positive parental and family environmental characteristics are positively related to indicators of children’s social development, and vice versa. Parke and Buriel [19] showed that some aspects of parenting such as emotional expressiveness, responsiveness, and support have been stipulated in a number of frameworks as mechanisms that enable children to acquire social skills. Anme and Segal [20] also demonstrated that global parenting quality was positively associated with children’s social development, including social competence, and communication skills.

In the present study, we assessed dimensions of parenting practices at age 2 as a predictor variables. The development of two-year-old children―the time period of age 2 is the starting point of our cohort study, is exploding in a number of aspects (e.g., motor functions, language skills, cognitive and emotional development). Additionally, when children reach two years of age, most parents experience a difficult time due to the first rebellious period, a normal stage in their child’s development. During children’s second year of life, they tend to react negatively against their parents, change mood easily, and resort to having temper tantrums [21]. Therefore, parenting practice at age 2 is obviously important for the child development. In the present study, we examined the dimensions of parenting practices that have an impact on specific dimensions of child social skills, in order to explore in more detail the potential early protective factors that can influence diverse developmental pathways in child social skills. To achieve these goals, we applied a group-based trajectory model with predictor variables [14].

In summary, we first sought to advance our understanding of children’s social skill development by identifying developmental trajectories of social skills during early childhood, from ages 2 to 5. Next, we investigated how parenting practices affect trajectory group membership. Although previous studies have already shown that better quality of parental care and family environment is associated with better development of child social skills, the present study sought to examine in detail the specific parenting factors that contribute to more sound developmental trajectories of social skills.

## Methods

### Participants

Data were drawn from a population-based study that constituted part of a nationwide ongoing longitudinal study in Japan [20, 22]. Sixty child day-care centers and 60 child night-care centers authorized by the government were asked to participate and cooperate, and 98 agreed to participate in this study at the beginning of the longitudinal project (cooperation rate 81.7%). The participants in this study consisted of boys and girls who attended child care centers and who were first assessed at the age of 2 by their parents and by nursery staff members. From 2000 on, data were collected with written informed consent in the winter of each year. All study participants gave written informed consent, and one of parents provided written informed consent on behalf of the children involved in the study. All research procedures were reviewed and approved by the institutional review board and ethics committee in University of Tsukuba. The baseline return rate was 74.6% for both parents and nursery staff, yielding information from 98 child day-care centers. Parents were surveyed regarding their parenting practices (e.g., parental involvement and social support for parenting), and nursery staff members evaluated the social skills development of each child in the facility, as well as aspects of parental child care quality. In order to control for ethnic and clinical background, children with a parent who was not born in Japan and children who did not speak Japanese as a native language, as well as children with disabilities, were excluded from the sample. After taking the above information into account, the final usable set of follow-up responses of homogeneous Japanese-speaking boys and girls totaled 1,055. As shown in Table 1, the distribution of boys and girls was fairly even. An average of .49 siblings lived in each household, with a range of zero to one (*SD* = .50). When children were first assessed, a majority (82.6%) were primarily living with both of their biological parents. The remaining children were living with their mother or father (14.8%), and 2.7% were living with other extended family members. In conducting the trajectory analysis, we did not include cases that were missing predictor or covariate information. Therefore, among the 1,055 possible participants, 1,000 for Cooperation, 997 for Self-Control, and 1,026 for Assertion were available because of missing predictor variables or covariates at 2 years of age. Of the total participants, 345–376 (32.7%-34.8%) provided social skills data on all four assessment points, 304–317 (28.8%-30.1%) on three, 192–220 (18.2%-20.9%) on two, and 161–173 (15.3%-16.4%) on only one. To test the effect of attrition bias in all four possible groups, we conducted a one-way ANOVA. There were no significant differences between children who did and did not participate continuously in terms of each domain of social skills at age 2: *F*(3, 996) = 2.06, *p* = .10, for Cooperation; *F*(3, 993) = 1.51, *p* = .21, for Self-Control; and *F*(3, 1022) = .91, *p* = .44, for Assertion. By means of multiple comparisons with Bonferroni correction, we also confirmed that there were no significant differences, so that the series of analyses in this study were conducted under a missing at random assumption.

**Table 1:** Descriptive statistics of all variables.

variables	*N*	*Mean*	*SD*	min	max
sex	1,055	0.49	0.50	0	1
sibling	1,055	0.49	0.50	0	1
*parental practices at age 2*					
2-year cognitive and emotional involvement	1,055	0.29	2.13	–16.24	2.19
2-year avoidance of punishment and restriction	1,055	0.14	0.86	–10.05	0.25
2-year social stimulation	1,055	0.06	1.68	–14.07	5.04
2-year social support on parenting	1,055	0.15	1.63	–12.55	1.19
*child social skills at ages 2 to 5*					
2-year cooperation	1,000	1.95	3.26	0	16
3-year cooperation	837	5.63	4.56	0	16
4-year cooperation	689	9.32	4.81	0	16
5-year cooperation	378	11.90	4.29	0	16
2-year self-control	997	6.19	3.97	0	16
3-year self-control	854	9.94	3.75	0	16
4-year self-control	698	12.35	3.36	0	16
5-year self-control	387	13.81	2.84	0	16
2-year assertion	1,026	13.28	2.70	4	16
3-year assertion	863	14.73	1.89	4	16
4-year assertion	701	15.04	1.81	5	16
5-year assertion	386	15.27	1.63	7	16

*Note*. Parenting variables were standardized due to different rating scales used for certain items.

### Measures

#### Social skills

Social skills were assessed by nursery staff members at four time points (ages 2, 3, 4, and 5). An adapted version of the Social Skills Questionnaire for Preschoolers (SSQ-P; [23]) was used as an index of observer ratings of child social skills at each age. Child social skill scores measured by the SSQ-P predict internalizing and externalizing behaviors longitudinally [23]. Because one of the most widely used social skill scales, the Social Skills Rating System (SSRS; [24]), which was used in the NICHD study (e.g., [25, 26]), should be applied to children aged 3 or over, for this study we used an adapted version of the SSQ-P, which is appropriate for children below the age of 3 years. The SSQ-P was originally designed to measure social skills from ages 4 to 6. In order to expand coverage to children at ages 2 and 3, we first analyzed whether any of the items were inappropriate for younger-aged children. After an exploratory factor analysis including the data from children aged 2 and 3, we selected 24 items from the original form (8 items for each subscale). The 24-item version included the same three subscales used in the SSRS: Cooperation (e.g., the child helps someone voluntarily), Self-Control (e.g., the child behaves him/herself if there is a need), and Assertion (e.g., the child initiates conversation with someone). Nursery staff members evaluated how often a social behavior occurred on a 3-point scale (0 = *never*, 1 = *sometimes*, 2 = *very often*). All subscales showed high internal reliability, ranging from .83–.94 for age 2, .85–.95 for age 3, .86–.95 for age 4, and .87–.96 for age 5 (Table 2). To confirm convergent validity, we computed correlations between the adapted version of the SSQ-P and the SSRS teacher form for preschoolers by using a subsample. The results indicated that the two scales were moderately positively correlated with each other, ranging from .25–.67 for age 2, .56–.75 for age 3, .73–.86 for age 4, and .75–.89 for age 5. Correlation between SSRS Cooperation and our scale’s Cooperation was somewhat low (*r* = .25), but this is probably because the SSRS preschoolers version should be applied to children aged 3 or over.

**Table 2:** Correlations between child social skills and all predictor variables.

	2-year cooperation		2-year self-control		2-year assertion		3-year cooperation		3-year self-control		3-year assertion		4-year cooperation		4-year self-control		4-year assertion		5-year cooperation		5-year self-control		5-year assertion	
sex (0 = girl, 1 = boy)	–.11	*	–.18	**	–.13	**	–.18	**	–.25	**	–.18	**	–.20	**	–.33	**	–.18	**	–.20	**	–.41	**	–.22	**
sibling (0 = no, 1 = one)	.01		.05		.04		.01		.03		–.02		.03		–.07		.03		.05		–.07		–.05	
2-year cognitive and emotional involvement	–.04		.03		.04		–.01		.07	*	.09	**	.00		.11	**	.05		.15	**	.24	**	.14	**
2-year avoidance of punishment and restriction	–.07		.00		.04		–.02		.02		.08	*	–.02		.06		.07		.09		.09		.13	*
2-year social stimulation	.00		.03		.03		.00		.05		.06		.05		.07		.07		.04		.12	*	.11	*
2-year social support on parenting	–.04		.01		.04		–.02		.01		.05		.05		.13	**	.10	**	.16	**	.23	**	.17	**
2-year cooperation	(.94)																							
2-year self-control	.64	**	(.91)																					
2-year assertion	.37	**	.55	**	(.83)																			
3-year cooperation	.56	**	.49	**	.40	**	(.95)																	
3-year self-control	.41	**	.65	**	.48	**	.64	**	(.91)															
3-year assertion	.23	**	.39	**	.67	**	.44	**	.59	**	(.85)													
4-year cooperation	.46	**	.45	**	.41	**	.66	**	.58	**	.47	**	(.95)											
4-year self-control	.34	**	.59	**	.48	**	.50	**	.77	**	.58	**	.69	**	(.93)									
4-year assertion	.25	**	.40	**	.66	**	.40	**	.55	**	.79	**	.55	**	.66	**	(.86)							
5-year cooperation	.39	**	.42	**	.42	**	.60	**	.61	**	.53	**	.78	**	.70	**	.56	**	(.96)					
5-year self-control	.33	**	.57	**	.50	**	.50	**	.76	**	.62	**	.64	**	.88	**	.65	**	.76	**	(.94)			
5-year assertion	.25	**	.42	**	.70	**	.40	**	.57	**	.80	**	.52	**	.65	**	.84	**	.63	**	.71	**	(.87)	

*Note*. * *p* < .05

** *p* < .01; for correlations between child social skills and child’s sex and presence of sibling, polychoric correlation coefficients were calculated.

Numbers in parenthesis show Cronbach’s alpha.

#### Parenting practices

To assess several parenting practices, including parental involvement, parental styles, and social support for parenting, we adapted the categories used by Anme and Segal [20]. This tool contains 12 items, with 6 self-report items rated by parents and 6 other-report items rated by nursery staff. It can be divided into four categories: cognitive and emotional involvement (4 items; e.g., reading books, singing songs together); avoidance of restriction and punishment (2 items, including parents being too harsh in disciplining and parents excessively controlling the child); social stimulation (3 items; e.g., visiting coeval friends’ houses, going to the park with the child); and social support for parenting (3 items; e.g., having someone to consult on child care, being supported in child care by someone). To form composite scores indicating greater or lesser quality of parenting, items were z-scored before the composite was computed because of the different rating scales used for certain items.

#### Covariates

We selected child’s sex and presence or absence of siblings in the family as covariates. First, several studies have indicated that the child’s sex has a strong influence on social skills development (i.e., girls > boys; [27, 28]). Moreover, the sibling relationship is likely to last longer than any other relationship in one’s lifetime and plays an integral part in the lives of families [29]. When a child has siblings, his or her interpersonal experiences are likely to increase, which may be positively associated with the child’s social skills development [30].

### Data Analysis Plan

We first calculated descriptive statistics, including means, standard deviations, range, and correlations for social skills scores at different ages. Developmental trajectories of social skills were estimated using a group-based trajectory model with predictors [14]. This method allowed us to identify separate subgroups among children aged 2 to 5, and to estimate both the membership probability of each trajectory and the effect of predictors on it. The software used to estimate trajectories was a SAS-based procedure “proc traj” [31]. A censored normal model was fitted to the data, because the data used to estimate the trajectories were based on psychometric scales with censoring at the scale minimum and maximum, and this censored normal trajectory modeling is considered to be well suited to accommodate a common feature of psychometric scale data [14]. Additionally, because this model is represented by not a single, but a mixture of the censored normal distributions, we have chosen to apply the censored normal model, not the ordinary normal model. Our selection of the optimal number of trajectory groups was basically based on the Bayesian information criteria (BIC). The model allows us to calculate posterior probabilities of group membership, which estimates each individual’s probability of belonging to each of the trajectory groups. A more comprehensive discussion of the technical details of this approach can be found elsewhere [14, 31]. After establishing the trajectories, we then sought to answer the next research question: How do parental and environmental factors predict children’s social skills trajectories? With covariates and parenting practices for trajectory group membership, the model allowed us to compute the effect of predictor variables on the probability of trajectory group membership. A multinomial logistic function was used to specify this probability function. By estimating a generalized model in which probability of trajectory group membership was conditional on individual characteristics and the trajectories themselves were jointly estimated, we avoided biases in statistical tests due to classification errors in trajectory group membership. In trajectory analysis, subjects with some missing longitudinal variables were included in the analysis, since these were assumed to be missing at random. Maximum likelihood estimation was used to estimate the model parameters.

## Results

### Descriptive Results

The means, standard deviations, and ranges for all predictor and outcome variables are provided in Table 1. Intercorrelations among child social skills and predictor variables are presented in Table 2. As the means shown in Table 1 indicate, there was a general trend for each child’s social skills to increase with age. The correlations among child social skill dimensions were moderately positive, both concurrently and across time. Table 2 also indicates that the polychoric correlations between child’s sex and social skill dimensions were always significantly negative, meaning that girls consistently showed higher social skills than boys. The polychoric correlations between the presence of siblings in the family and social skill dimensions in Table 2 were not significant in any case.

### Developmental Trajectories

**Fig 1:**
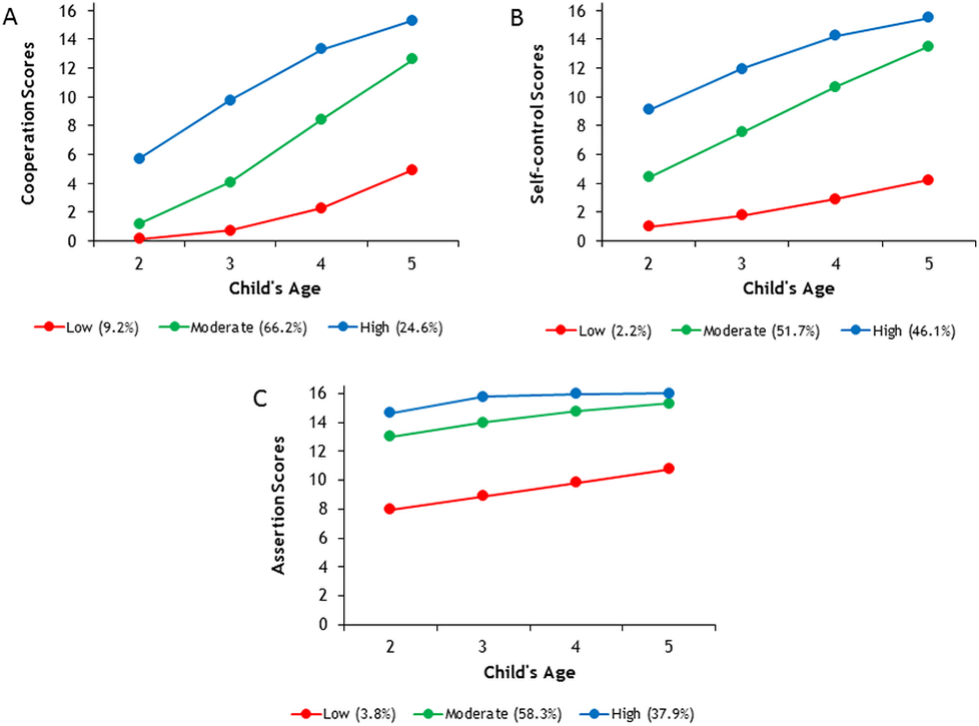
Developmental trajectories of social skill dimensions from ages 2–5. (A) Developmental trajectories on Cooperation dimension. (B) Developmental trajectories on Self-control dimension. (C) Developmental trajectories on Assertion dimension.

We identified groups of children showing distinct longitudinal profiles of social skills over time using a group-based trajectory model with predictor variables. In order to determine the optimal number of trajectories needed to describe the transition of social skills development from 2 to 5 years of age for this sample, we fitted models with two, three, four, and five profiles, based on BIC. For Cooperation, the BIC was –6734.30 for two trajectories, –6712.30 for three, –6719.87 for four, and –6724.92 for five. For Self-Control, the BIC was –7349.24 for two trajectories, –7290.40 for three, –7277.74 for four, and –7293.24 for five. For Assertion, the BIC was –4990.72 for two trajectories, –4945.61 for three, –4945.56 for four, and –4954.23 for five. By using the BIC criterion, a three-group model was selected for Cooperation and four-group models for Self-Control and Assertion. However, although the four-group models for Self-Control and Assertion showed greater BICs, the three-group models for these two dimensions already subdivided modest size groups with higher levels of child social skills into smaller groups that did not differ substantively [32]. Thus, for all three dimensions of child social skills, the model with three trajectories was identified as the best fitting and most parsimonious. Further, in order to judge objectively the adequacy of the final three-group models, the average posterior probabilities for each group (i.e., the mean group assignment probabilities, conditional on assignment by the maximum likelihood probability rule) were calculated. The average posterior probability of group membership measures the probability that an individual with a specific profile belongs to each of the model’s trajectory groups. For Cooperation, group 1 average posterior probability = .81, group 2 = .85, and group 3 = .83; for Self-Control, group 1 = .87, group 2 = .85, and group 3 = .86; and for Assertion, group 1 = .88, group 2 = .85, and group 3 = .78. In all cases, the average posterior probabilities were greater than .70, a value that Nagin [14] assumed to imply a good model fit to the data. As presented in the panel A of Fig 1, the three trajectories for Cooperation from ages 2 to 5 and the estimation of the proportion of the sample following each trajectory were as follows: low (9.2%), moderate (66.2%), and high (24.6%) groups. The low group followed a trajectory reflecting a slight increase later in the preschool age. The moderate group showed the sharpest increase, moving from almost the same level as the low group at age 2 to the level of the high group by age 5. The high group followed a trajectory reflecting a linear increase as they grew. The panel B in Fig 1 depicts the three trajectory groups of Self-Control, along with the estimated proportion of children in each group. The low group (2.2%) included children with lower levels of this dimension. The moderate group (51.7%) comprised approximately half of the children. The high group (46.1%) included children who showed higher levels during early childhood. All three trajectories increased linearly from ages 2 to 5. The panel C in Fig 1 illustrates the trajectories on the Assertion dimension for low (3.8%), moderate (58.3%), and high (37.9%) groups. For the low group, the trends can be described as a linear growing trajectory, whereas for the moderate and high groups, the trends were stable over time and remained at a relatively high level.

### Predictors for Social Skills Trajectory Membership

We next examined the contributions of all predictor variables in distinguishing group memberships for child social skills trajectories. We examined the relative contributions of four parenting practices at age 2: cognitive and emotional involvement, avoidance of punishment and restriction, social stimulation, and social support for parenting. The child’s sex and the presence of siblings were entered as covariates in order to control for these effects on child social skills development. In the multinomial logistic analysis, all groups were compared to the low group (Table 3). When all predictors were entered together in the models, several child and familial characteristics emerged as significant factors. The child’s sex most consistently distinguished social skills group membership: Being a girl was associated with higher levels of social skills development (*B* = –1.36, *p* < .01 for Cooperation; *B* = –1.88, *p* = .01 for Self-Control; *B* = –1.56, p < .01 for Assertion) and the child’s sex effect gave some indication of distinguishing between the low and moderate group for Assertion (*B* = –1.02, *p* = .04). The second strongest association with differential trajectory memberships was cognitive and emotional involvement. This tended to increase the likelihood of membership to the moderate and high groups (*B* = .19 and .14, *p* = .03 and .08, respectively). For Assertion, cognitive and emotional involvement also distinguished between the low group and both the moderate and high groups in the expected direction (*B* = .19 and .15, *p* = .03 and .07, respectively). Social stimulation significantly distinguished between the low and both the moderate and high groups on the Self-Control dimension (*B* = .30 and .29, *p* = .03 and .03, respectively). For Assertion, social stimulation showed some indication of distinguishing the low from the moderate (*B* = .33, *p* = .01) and from the high groups (*B* = .27, *p* = .03). Finally, within a multivariate framework, the presence of siblings and social support for parenting did not distinguish between any pairs of trajectory groups.

**Table 3:** Multinomial logistic analyses for predicting social skills trajectory group memberships by covariates and four categories of parenting.

Predictor Variables	Low Vs. Moderate			Low Vs. High		
	*B*	Wald Test	*p*	*B*	Wald Test	*p*
*Cooperation*						
Sex	–.51	–1.29	.20	–1.36	–3.53	.00
Sibling	–.09	–.24	.81	.20	.56	.57
2-year cognitive and emotional involvement	.19	2.22	.03	.14	1.76	.08
2-year avoidance of punishment and restriction	.22	1.45	.15	.02	.16	.87
2-year social stimuli	.01	.13	.89	.04	.43	.67
2-year social support on parenting	–.06	–.60	.55	–.05	–.53	.60
*Self-Control*						
Sex	–.79	–1.15	.25	–1.88	–2.78	.01
Sibling	–.52	–.83	.41	–.23	–.37	.71
2-year cognitive and emotional involvement	.06	.58	.56	.16	1.47	.14
2-year avoidance of punishment and restriction	.20	1.29	.20	.14	.90	.37
2-year social stimuli	.30	2.15	.03	.29	2.12	.03
2-year social support on parenting	.20	1.47	.14	.19	1.41	.16
*Assertion*						
Sex	–1.02	–2.01	.04	–1.56	–3.13	.00
Sibling	.08	.19	.85	.16	.38	.70
2-year cognitive and emotional involvement	.19	2.24	.03	.15	1.84	.07
2-year avoidance of punishment and restriction	.33	2.59	.01	.27	2.20	.03
2-year social stimuli	.09	.92	.36	.14	1.33	.18
2-year social support on parenting	–.13	–1.13	.26	.03	.26	.79

## Discussion

In the present study, we analyzed longitudinal data collected throughout Japan in order to describe the patterns of consistency and change in the development of social skills for preschoolers. We also examined the links between the developmental patterns of children’s social skills and parenting practices at an early developmental phase.

Although there have been several longitudinal studies and reviews of the course of prosocial development during the preschool period [10, 27, 28], few longitudinal studies have focused on the specific course of social skills. The present study provided three important contributions to the study of preschoolers’ social skills development. The first concerns the developmental profiles of preschoolers’ social skills. Hay [28] mentioned that it is during the preschool period that children learn how not to be good, quoting a phrase in Machiavelli’s *The Prince*: “A prince must learn how not to be good.” However, from a socialization theory perspective [10], preschool aged children learn how to be good with age; namely, they show an increase in the frequency of prosocial behaviors. The present results support this perspective. Children’s social skills demonstrated an upward trend on every dimension during the preschool period of ages 2 to 5 (Table 1). Our results indicate that even during the preschool period, children learn and build their social skills, which are the very foundations of prosocial behaviors.

Our second contribution is the identification of three developmental trajectories for each of the three sub-dimension of social skills, low, moderate, and high, using group-based trajectory models. As hypothesized, these descriptions of longitudinal profiles were similar to prior research that examined trajectories of children’s prosocial development [7, 12, 13, 15]. In addition, a small percentage of children in the present study showed low levels of social skills from ages 2 to 5. In previous studies that examined the trajectories of prosocial behaviors from childhood to early adolescence, the percentages of low social skills groups ranged from 7% to 53%; however, in the present study, 10% or less of preschool aged children displayed a chronically low level of social skills. This smaller percentage suggests that we may be able to identify a group of children in in need of social skills training. It also suggests that we need to identify potential protective factors that distinguish children in a chronically low group from those who display moderate or high level skills during early childhood.

Our third, and most important, finding was the links between parenting practices at age 2 and child social skills development. Past research has shown that positive parenting can predict later sound social development of children [18, 19, 20], and negative parenting can also predict children’s subsequent problem behaviors and interpersonal conflict [6, 17]. As shown in Table 2, each parenting practice was basically positively associated with children’s social skill subscale scores. It is interesting to note that correlations of parenting practices at age 2 and children’s social skills scores at age 2 are small but increase as the children get older. This indicates that parenting practices at age 2 may not confer a short-term benefit to children, but rather bear fruit over time.

We conducted multinomial logistic analyses to examine to what extent each dimension of parenting practices affects child social skills development. The results indicate that cognitive and emotional involvement tends to stimulate the development of Cooperation and Assertion, that avoidance of restriction and punishment encourages a child to increase Assertion, and that social stimulation drives development of Self-control. These results suggest that child social skills development is *domain-specifically* associated with parenting practices. That is, Cooperation (as well as Assertion) was enhanced by cognitive and emotional involvement, Self-control by social stimulation, and Assertion by avoidance of restriction and punishment. It is reasonable to mention that treating a child with emotional warmth helps the child acquire skills for acting cooperatively to avoid conflict with others and to interact well with others. Without excessive restrictions, a child might become more decidedly assertive. Social stimulation from the outside world would induce a child to learn how to behave outside the home, and about the potentials for good behaviors and for what is socially acceptable including the need for self- regulation. The discovery of domain-specific links between child social skills development and parenting practices implies that a specific dimension of parenting practices can be an efficient intervention for social skills deficits, depending on which aspect of social skills a child has problems with.

The present study found that social support for parenting was positively correlated with subsequent child social skills development; however, social support for parenting did not have a significant positive effect on predicting the likelihood of children belonging to higher social skills trajectories. This pattern of results suggests that the observed relation between social support for parenting and child social skills, as shown in Table 2, may be spurious. Thus, even in cases where parents feel a lack of social support for parenting, this could be compensated by other types of appropriate parenting practices.

Additionally, in order to test how global parenting quality influences child social skills development, we conducted a supplemental analysis, a multinomial logistic analyses by entering a total score of all four categories for global parenting child care quality. The result shown in Table 4 revealed that global parenting quality consistently and significantly distinguished between the low group and both the moderately low and high groups (for Cooperation, *B* = .11, *p* < .01, *B* = .09, *p* = .01, respectively; for Self-Control, *B* = .14, *p* < .01, *B* = .17, *p* < .01, respectively; for Assertion, *B* = .09, *p* < .01, *B* = .13, *p* < .01, respectively), indicating that global quality of parental child care fertilizes all three dimensions of child social skills.

**Table 4:** Multinomial logistic analyses for predicting social skills trajectory group memberships by covariates and global quality of parenting.

Predictor Variables	Low Vs. Moderate			Low Vs. High		
	*B*	Wald Test	*p*	*B*	Wald Test	*p*
*Cooperation*						
Sex	–.78	–1.52	.13	–1.62	–3.18	.00
Sibling	–.55	–1.09	.28	–.30	–.58	.56
2-year global parenting quality	.11	3.22	.00	–.09	2.62	.01
*Self-Control*						
Sex	–.86	–1.25	.21	–1.95	–2.90	.00
Sibling	–.50	–.82	.42	–.24	–.39	.70
2-year global parenting quality	.14	2.90	.00	.17	3.70	.00
*Assertion*						
Sex	–.82	–1.70	.09	–1.39	–2.88	.00
Sibling	.09	.21	.84	.19	.45	.65
2-year global parenting quality	.09	2.92	.00	.13	3.59	.00

We found the children’s sex to be a fairly robust discriminative factor. Several studies have established that the level of social skills is higher in girls than in boys [27, 28]. Zahn-Waxler, Robinson, and Emde [32] also reported that girls were more likely than boys to show signs of empathy, one dimension of prosocial behaviors, even in the second year of life. The present results indicate that girls are already more prosocial than boys in early childhood. Hay [28] stated that gender difference becomes increasingly apparent after infancy, and that females have been reported to show more prosocial traits than males do throughout the life span. With regard to the sibling effect, relatively little attention has been devoted to the role of siblings and their impact on one another’s development, in comparison to the wealth of studies on parent-child relationships (Howe & Recchia, 2006). However, the presence of any siblings plays an important role in the development of children’s understanding of others’ minds, namely their understanding of emotions, thoughts, intentions and beliefs [33]. When a child has siblings, this is likely to increase the interpersonal experiences regardless of whether the child is at home or outside the home. Compared to only children, children with siblings should have more social interactions [30], which may accelerate the development of their social skills. Thus, it is somewhat surprising that the presence of siblings had no differentiating effect on trajectory group membership. The polychoric correlations between the presence of any siblings in the household and social skill dimensions were not significant either, indicating that the structural variable of having a sibling generally did not exert a favorable impact on child social skills development. To clarify further the effect of siblings on child social skills development, future research should take into account the quality of sibling interaction, the frequency of sibling conflict, birth order, and gender.

Several limitations of the present study need to be addressed. First, parenting practices can be based on a variety of elements, and the four dimensions we used in this study are not comprehensive. For instance, laxness, poor supervision, inconsistent discipline, and interparental conflict are negatively associated with child social skills development [34, 35]. Second, although we investigated sex and the presence of siblings, there may be other significant background variables that differentiate among the trajectory groups, especially as potential risk factors for the chronically low social skill group, for example, parental income and education. Third, another limitation concerns the possibility of a reciprocal relationship between parenting practice quality and child social skill development. Because parenting practice quality does not always have a unidirectional pathway to child social skills development, more studies are needed to examine these bi-directional associations more closely. Fourth and finally, participants were likely to be homogeneous because all of the samples were Japanese. Generalizability therefore remains to be confirmed through research on more heterogeneous samples.

Despite these limitations, this study has contributed to the understanding of how parenting factors are linked to child social skills development in the preschool years. The present results are consistent with those of previous studies which have demonstrated that the quality of parental behaviors consistently predicts subsequent social competence and problem behaviors [6, 17]. Therefore, we confirm the important role of the parental childcare effect in child social development. Further, the current findings have implications for early childhood practice and policy, for expanded access to social skills programs by preschoolers, and for improvements in parental and family environments.

## References

[1] Gresham FM, Elliott SN (1984) Assessment and Classification of children’s social skills. A review of methods and issues. School Psychol Rev 13: 292–301.

[2] Arnold DH, Kupersmidt JB, Voegler-Lee ME, Marshall N (2012) The association between preschool children’s social functioning and their emergent academic skills. Early Child Res Q, 27: 376–386. 23002324 10.1016/j.ecresq.2011.12.009 PMC3445416

[3] Veenstra R, Lindenberg S, Oldehinkel AJ, De Winter AF, Verhulst FC, Ormel J (2008) Prosocial and antisocial behavior in preadolescence: teachers’ and parents’ perceptions of the behavior of girls and boys. Int J Behav Dev, 32: 243–251.

[4] Chan D, Ramey S, Ramey C, Schmitt N (2000) Modeling intraindividual changes in children’s social skills at home and at school: A multivariate latent growth approach to understanding between-settings differences in children’s social skill development. Multivar Behav Res, 35: 365–396. 10.1207/S15327906MBR3503_04 26745336

[5] Berry D, O’Connor E (2010) Behavioral risk, teacher–child relationships, and social skill development across middle childhood: A child-by-environment analysis of change. J Appl Dev Psychol, 31: 1–14.

[6] Vazsonyi AT, Huang L (2010) Where self-control comes from: On the development of self-control and its relationship to deviance over time. Dev Psychol, 46: 245–257. 10.1037/a0016538 20053021

[7] Luengo KBP, Pastorelli C, Eisenberg N, Zuffianò A, Caprara GV (2013) The development of prosociality from adolescence to early adulthood: the role of effortful control. J Pers, 81: 302–312. 10.1111/jopy.12001 22924862

[8] Stright AD, Gallagher KC, Kelley K (2008) Infant temperament moderates relations between maternal parenting in early childhood and children’s adjustment in first grade. Child Dev, 79: 186–200. 10.1111/j.1467-8624.2007.01119.x 18269517

[9] Brownell CA (2013) Early development of prosocial behavior: Current perspectives. Infancy, 18: 1–9. 25632273 10.1111/infa.12004 PMC4306462

[10] Eisenberg N, Fabes RA, Spinrad TL (2006) Prosocial development In Eisenberg N., Damon W., & Lerner R. M. (Eds.), Handbook of child psychology: Vol. 3, Social, emotional, and personality development (6th ed., pp. 646–718). Hoboken, NJ: Wiley.

[11] Caplan MZ (1993) Inhibitory influences in development: The case of prosocial behavior In: Hay DF, & Angold A, editors. Precursors and causes in development psychopathology. New York: Wiley pp. 169–198.

[12] Kokko K, Tremblay RE, Lacourse E, Nagin DS, Vitaro F (2006) Trajectories of prosocial behavior and physical aggression in middle childhood: Links to adolescent school dropout and physical violence. J Res Adolescence, 16: 403–428.

[13] Nantel-Vivier A, Kokko K, Caprara GV, Pastorelli C, Gerbino M, Paciello M, et al (2009) Prosocial development from childhood to adolescence: A multi-informant perspective with Canadian and Italian longitudinal studies. J Child Psychol Psyc, 50: 590–598. 10.1111/j.1469-7610.2008.02039.x 19207631

[14] Nagin DS (2005) Group-based modeling of development Cambridge, MA: Harvard University Press.

[15] Côté S, Tremblay RE, Nagin DS, Zoccolillo M, Vitaro F (2002) The development of impulsivity, fearfulness, and helpfulness during childhood: patterns of consistency and change in the trajectories of boys and girls. J Child Psychol Psyc, 43: 609–618. 10.1111/1469-7610.00050 12120857

[16] Lamont A, Van Horn ML (2013) Heterogeneity in parent-reported social skill development in early elementary school children. Soc Dev, 22: 384–405.

[17] Tichovolsky MH, Arnold DH, Baker CN (2013) Parent predictors of changes in child behavior problems. J Appl Dev Psychol, 34: 336–345. 10.1016/j.appdev.2013.09.001 PMC3859377 24347757

[18] Repetti RL, Taylor SE, Seeman TS (2002) Risky families: Family social environments and the mental and physical health of the offspring. Psychol Bull, 128: 330–366. 11931522

[19] Parke RD, Buriel R (1998) Socialisation in the family: Ethnic and ecological perspective In: Damon W, series editor and Eisenberg N, volume editor, Handbook of Child Psychology. Volume 3: Social, emotional and personality development (5th edition). New York: Wiley, pp. 463–552.

[20] Anme T, Segal UA (2004) Implications for the development of children in over 11 hours of centre-based care. Child Care Hlth Dev, 30: 345–352. 10.1111/j.1365-2214.2004.00429.x 15191425

[21] Shaw DS, Bell RQ (1993) Developmental theories of parental contributors to antisocial behavior. J Abnorm Child Psychol, 21: 493–518. 8294650 10.1007/BF00916316

[22] Anme T, Segal UA (2007) Implications of Japan’s center-based night care: A one-year follow-up. Early Child Educ J, 35: 293–299.

[23] Takahashi Y, Okada K, Hoshino T, Anme T (2008) Social skills for preschoolers: Stability of factor structures and predictive validity from a nationwide cohort study in Japan. Jap J Educ Psychol, 56: 81–92.

[24] Gresham FM, Elliott SN (1990) Social skills rating system manual Circle Pines, MN: American Guidance Service.

[25] NICHD Early Child Care Research Network (2002) Early child care and children’s development prior to school entry. Am Educ Res J, 39: 133–164.

[26] NICHD Early Child Care Research Network (2004) Father’s and mother’s parenting behavior and beliefs as predictors of child social adjustment in the transition to school. J Fam Psychol, 18: 628–638. 15598168 10.1037/0893-3200.18.4.628

[27] Eisenberg N, Fabes RA (1998) Prosocial development In: Damon W, series editor, and Eisenberg N, volume editor, Handbook of child psychology: Vol. 3 Social, emotional, and personality development New York: Wiley pp. 701–778.

[28] Hay DF (1994) Prosocial development. J Child Psychol Psyc, 35: 29–71. 10.1111/j.1469-7610.1994.tb01132.x 8163628

[29] Howe N, Recchia H (2006) Sibling relations and their impact on children’s development In: Tremblay RE, Barr RG, Peters RD, Boivin M, editors. Encyclopedia on Early Childhood Development [online]. Montreal, Quebec: Centre of Excellence for Early Childhood Development Available at: http://www.child-encyclopedia.com/documents/Howe-RecchiaANGxp.pdf. Accessed [2015-04-30].

[30] Downey DB, Condron DJ, Yucel D (2015) Number of siblings and social skills revisited among American fifth graders. J Fam Issues, 36: 273–396.

[31] Jones BL, Nagin DS (2007) Advances in group-based trajectory modeling and SAS Procedure for estimating them. Sociol Method Res, 35: 254–571.

[32] Zahn-Waxler C, Robinson J, Emde R (1992) The development of empathy in twins. Dev Psychol, 28: 1038–1047.

[33] Dunn J (2002) Sibling relationships In: Smith PK, Hart CH, editors. Blackwell handbook of childhood social development. Malden, Mass: Blackwell Publishing pp. 223–237.

[34] Arnold DS, O’Leary SG, Wolff LS, Acker MM (1993) The Parenting Scale: A measure of dysfunctional parenting in discipline situations. Psychol Assessment, 5: 137–144.

[35] Krishnakumar A, Buehler C (2000) Interparental conflict and parenting practices: A meta-analysis. Fam Relat, 49: 25–44.

